# N6-methyladenosine as a potential epitranscriptomic immune rheostat during SARS-CoV-2 infection

**DOI:** 10.3389/fimmu.2026.1878455

**Published:** 2026-09-02

**Authors:** Xianfeng Hui, Shuoxiang Gao, Shihuan Ding, Mengyi Li, Xiaowei Tian

**Affiliations:** 1 Department of Immunology, School of Basic Medical Sciences, Henan Medical University, Xinxiang, China; 2 Department of Pathogenic Biology, School of Basic Medical Sciences, Henan Medical University, Xinxiang, China

**Keywords:** antiviral immunity, epitranscriptomic regulation, N6-methylAdenosine (m6A), SARS-CoV-2, type I interferon signaling

## Abstract

N6-methyladenosine (m^6^A), the most abundant internal RNA modification in eukaryotic cells, has emerged as a critical regulator of antiviral host defense. Increasing evidence indicates that m^6^A functions beyond conventional post-transcriptional regulation by dynamically coordinating the magnitude, timing, and duration of immune responses during viral infection. In SARS-CoV-2 infection, dysregulated antiviral immunity is characterized by delayed interferon activation together with sustained inflammatory responses, suggesting the existence of regulatory mechanisms that continuously calibrate immune signaling rather than simply switching it on or off. In this review, we propose the concept of m6A as a potential “epitranscriptomic immune rheostat,” representing a conceptual framework in which m6A may fine-tune antiviral immunity through coordinated regulation of RNA stability, translational efficiency, and transcript turnover. We summarize how m^6^A shapes multiple layers of innate immune responses, including pattern recognition receptor sensing, type I interferon signaling, inflammatory buffering, and immune resolution. We further discuss emerging evidence suggesting that SARS-CoV-2 infection is associated with dynamic modulation of the m^6^A regulatory machinery and viral RNA methylation landscapes, while emphasizing that many mechanistic insights remain to be experimentally validated in SARS-CoV-2 models. In addition, we highlight the interplay between m^6^A regulation and immunometabolic remodeling, while distinguishing direct SARS-CoV-2 evidence from findings derived from other viral systems or broader m^6^A biology. Selected comparisons with Influenza A virus are discussed as complementary evidence to explore potentially conserved principles of m^6^A-mediated immune regulation among RNA viruses rather than as direct evidence for SARS-CoV-2 infection. Finally, we discuss the therapeutic implications of targeting the m^6^A regulatory network to recalibrate immune responses with temporal and cellular precision, while noting that current m^6^A-targeting strategies remain at the preclinical proof-of-concept stage and require further evaluation regarding specificity, safety, and translational feasibility. Collectively, this review provides a hypothesis-driven conceptual framework for understanding how m^6^A integrates RNA fate control with antiviral immunity and immunopathology during SARS-CoV-2 infection.

## 1 Introduction

Since its emergence in late 2019, the outbreak of SARS-CoV-2 has led to the global COVID-19 pandemic, posing an unprecedented challenge to public health systems worldwide (1). Although the deployment of vaccines and antiviral therapies has significantly reduced disease burden, immune dysregulation remains a defining feature of severe cases and a major determinant of disease progression and mortality. Accumulating evidence indicates that the immune phenotype of COVID-19 is neither characterized by simple immunosuppression nor by uniform hyperactivation, but rather by a profound imbalance (2). On one hand, the early Type I Interferon response is often impaired or delayed, compromising the initial control of viral replication (3, 4). On the other hand, as infection progresses, inflammatory responses become progressively amplified, frequently culminating in excessive immune activation typified by cytokine storm, which contributes to tissue damage and multi-organ dysfunction (5, 6).

This characteristic pattern of “early insufficiency followed by late hyperactivation” highlights a critical principle: the effectiveness of antiviral immunity depends not merely on its magnitude, but on its precise temporal and spatial regulation (7). In this context, the host must maintain a dynamic balance between efficient viral clearance and the prevention of immunopathology, rather than simply maximizing or suppressing immune responses (2). Notably, SARS-CoV-2 has evolved multiple strategies to perturb this balance, including delaying innate immune activation, antagonizing interferon signaling pathways, and modulating the amplitude of inflammatory responses (8). These strategies enable the virus to evade rapid immune clearance while simultaneously creating a permissive environment for replication.

In recent years, the field of epitranscriptomics has provided a new conceptual framework for understanding virus-host interactions (9). In addition to m^6^A, RNA modifications such as 5-methylcytosine (m^5^C), pseudouridine (Ψ), and adenosine-to-inosine (A-to-I) RNA editing have also been implicated in regulating viral replication and host antiviral immunity (10). Nevertheless, m^6^A is distinguished by its dynamic reversibility and its broad regulatory roles in both viral RNA metabolism and host immune signaling, making it particularly suitable for exploring the concept of the epitranscriptomic immune rheostat. Among various RNA modifications, N6-methyladenosine (m^6^A) is the most abundant internal modification in eukaryotic mRNA and has emerged as a critical layer of gene regulation due to its reversible and dynamic nature (11). Growing evidence suggests that m^6^A not only regulates RNA stability, splicing, and translation, but also contributes to the regulation of antiviral immune responses (12, 13). In particular, m^6^A exhibits pronounced temporal and dose-dependent effects in modulating pattern recognition receptor signaling, interferon production, and inflammatory gene expression (14). For example, m^6^A modification of key antiviral transcripts, including IFNB1 and IRF7, can influence their stability and translational efficiency, thereby shaping the magnitude and kinetics of type I interferon responses. Likewise, m^6^A-dependent regulation of inflammatory mediators such as IL-6 has been implicated in controlling the persistence and resolution of inflammatory signaling. These representative examples illustrate how m^6^A may function as a regulatory rheostat that continuously adjusts immune outputs across different stages of infection, supporting its conceptualization as an epitranscriptomic immune rheostat rather than a simple molecular switch.

Based on these observations, we propose a hypothesis-driven conceptual framework in which m^6^A functions not as a binary “on/off” switch, but rather as a tunable “immune rheostat (15)”. Mechanistically, m^6^A may dynamically shape the magnitude, timing, and duration of antiviral immune responses by fine-tuning the stability, translational efficiency, and expression kinetics of immune-related transcripts (16). In the context of SARS-CoV-2 infection, emerging evidence suggests that m^6^A-mediated regulation may influence host immune responses and viral replication; however, many aspects of this proposed rheostatic function remain to be experimentally validated in SARS-CoV-2 models (17).

In this review, we systematically summarize the molecular mechanisms by which m^6^A has been reported to regulate host antiviral immunity, with a particular focus on its potential role as an immune rheostat in innate immune activation, inflammatory regulation, and immunometabolic reprogramming. We further discuss emerging evidence from other RNA viruses, such as Influenza A virus, to explore potentially conserved regulatory principles while clearly distinguishing these findings from direct SARS-CoV-2 evidence. Finally, we highlight the therapeutic potential and challenges of targeting the m^6^A regulatory system for antiviral intervention.

## 2 Molecular architecture of the m^6^A immune rheostat

### 2.1 What defines an immune rheostat

An immune rheostat is a regulatory mechanism that continuously calibrates immune responses rather than operating as a binary on/off switch. Conceptually, such a system should exhibit three defining properties: quantitative tunability, bidirectional regulation, and temporal plasticity. By dynamically controlling RNA stability, translation, and transcript turnover, m^6^A has the potential to fulfill these criteria, supporting the hypothesis that m6A may function as an epitranscriptomic regulator of antiviral immune homeostasis.

Taken together, these proposed characteristics provide an operational framework for distinguishing an m6A-mediated immune rheostat from conventional post-transcriptional regulation. Rather than simply altering RNA fate, m^6^A has the potential to enable quantitative, bidirectional, temporally dynamic, and cell type-specific regulation of immune responses, thereby providing a mechanistic framework for precisely calibrating antiviral immunity.

Experimentally, these defining features can be evaluated by determining whether perturbation of m6A regulators quantitatively alters immune outputs, exhibits stage-dependent effects, supports bidirectional regulation under distinct conditions, and produces cell type-specific immune phenotypes.

### 2.2 Dynamic regulatory network of the m6A machinery

The m^6^A modification system comprises three functional modules-writers, erasers, and readers-forming a highly plastic and reversible epitranscriptomic regulatory network (18, 19). The methyltransferase complex, with METTL3 as its catalytic core, cooperates with METTL14 and adaptor proteins such as WTAP and VIRMA to deposit methyl groups onto adenosine residues within the conserved DRACH motif of RNA (20). In contrast, the demethylases FTO and ALKBH5 remove these modifications, enabling dynamic remodeling of the m^6^A landscape (11). Functionally, YTH domain-containing proteins (e.g., YTHDF1/2/3 and YTHDC1) act as “effectors” by recognizing m^6^A-modified transcripts and recruiting distinct RNA processing or decay machineries, thereby translating chemical marks into specific biological outcomes (21). Together, this modular architecture provides the molecular basis for rapid and reversible regulation of RNA fate and is therefore well suited to support rheostatic immune regulation.

**Figure 1:**
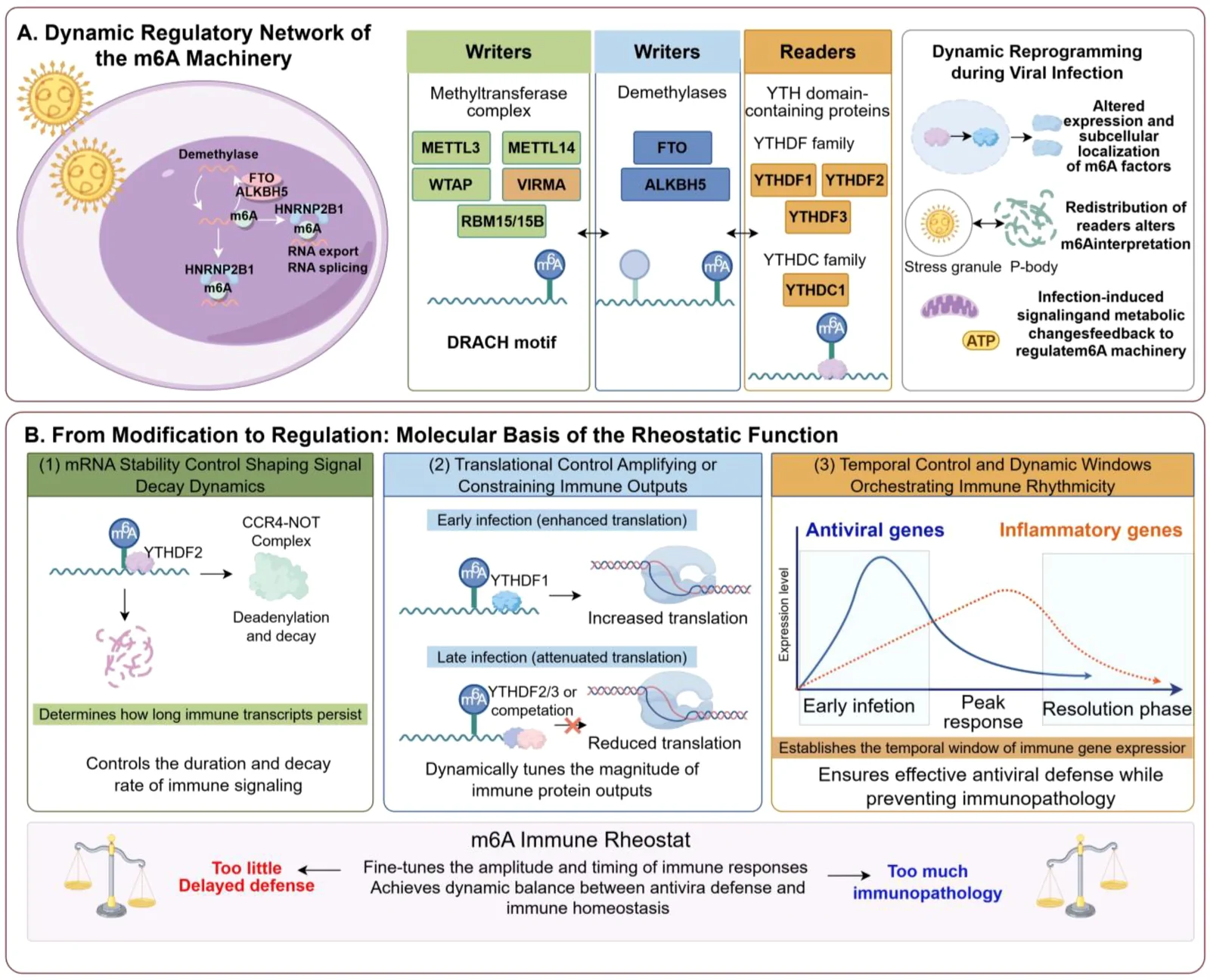
Molecular architecture of the m^6^A immune rheostat. This figure illustrates the concept of the m6A-mediated immune rheostat. It summarizes the dynamic regulatory network of m6A “writers,” “erasers,” and “readers,” and their reprogramming during viral infection **(A)**. Mechanistically, m6A controls RNA stability, translation, and temporal expression of immune-related transcripts, thereby fine-tuning antiviral and inflammatory responses **(B)**. Together, these multilayered regulatory processes enable m6A to function as a molecular rheostat that balances effective antiviral defense with prevention of immunopathology.

During viral infection, growing evidence indicates that this regulatory network undergoes extensive dynamic reprogramming (22). First, both the expression levels and subcellular localization of m^6^A enzymes can rapidly shift in response to infection. For instance, enhanced nuclear methyltransferase activity or redistribution to the cytoplasm can reshape the epitranscriptomic profiles of both viral RNA and host immune transcripts (23). Second, the relocalization of reader proteins across cellular compartments-such as their enrichment in stress granules or P-bodies-can alter the “interpretation” of m^6^A marks, enabling functional switching between translation enhancement and mRNA decay (23). Furthermore, infection-induced signaling pathways, including cellular stress responses and metabolic alterations, can feed back to regulate the activity of m^6^A machinery, forming a closed-loop regulatory circuit of “modification-signaling–remodification (24).” These features provide a mechanistic basis through which m^6^A may contribute to the dynamic tuning of immune responses (**Figure 1A**).

### 2.3 From modification to regulation: molecular basis of the immune rheostat

The ability of m^6^A to function as an “immune rheostat” arises from its capacity to continuously modulate RNA fate, rather than acting through unidirectional or binary regulatory mechanisms (25). By coordinately governing mRNA stability, translational efficiency, and expression kinetics, m^6^A establishes a multilayered regulatory framework that precisely calibrates the magnitude, timing, and duration of immune signaling outputs (21). This integrated mode of control enables dynamic tuning of antiviral responses, ensuring both effective pathogen restriction and the prevention of immunopathology (**Figure 1B**).

#### 2.3.1 mRNA stability control: shaping signal decay dynamics

m^6^A regulates transcript stability predominantly through the recruitment of YTHDF2 and associated RNA decay machineries, including the CCR4-NOT deadenylase complex, thereby promoting deadenylation and subsequent degradation of target mRNAs (26). This regulatory axis is particularly critical in antiviral immunity, where immune responses must be rapidly activated yet tightly resolved to prevent persistent inflammation and tissue damage. Importantly, the positional distribution of m^6^A marks-whether within the 3′ UTR or coding sequence-together with the selective engagement of reader proteins, determines the efficiency and kinetics of transcript turnover (19). Consequently, m^6^A-dependent regulation extends beyond binary control of gene expression to govern transcript persistence, thereby shaping the duration and decay dynamics of immune signaling at a systems level. This decay-centered mechanism positions m^6^A as a key determinant of immune signal resolution, rather than merely its initiation.

#### 2.3.2 Translational control: amplifying or constraining immune outputs

Beyond its role in transcript stability, m^6^A critically regulates protein synthesis by enhancing ribosome recruitment and translation initiation efficiency. Reader proteins such as YTHDF1 selectively recognize m^6^A-modified transcripts and promote the assembly of translation initiation complexes, thereby markedly increasing translational output (27, 28). During early antiviral responses, this mechanism has been reported to facilitate the rapid production of antiviral effectors and signaling intermediates, enabling an immediate and robust host response (26). In later stages, however, functional redistribution or competitive interactions among m^6^A reader proteins can attenuate translation, contributing to the controlled resolution of immune activation and preventing excessive inflammatory responses (29). In this context, m^6^A operates as a “tunable amplifier” at the level of protein expression, dynamically modulating the magnitude and timing of immune outputs.

#### 2.3.3 Temporal control and dynamic windows: orchestrating immune rhythmicity

Collectively, available evidence suggests that m^6^A may integrate the above mechanisms to achieve precise temporal regulation of immune responses. By promoting rapid translation of key immune genes at early stages of infection and accelerating their degradation during resolution phases, m^6^A establishes a defined temporal window of gene expression. This “rapid-onset, controlled-off” dynamic ensures that immune responses are sufficiently robust to suppress viral replication while avoiding prolonged activation that could lead to immunopathology (30). Moreover, the differential distribution of m^6^A modifications across transcripts enables functional specialization at different stages of the immune response-for instance, prioritizing antiviral gene expression while temporally restricting sustained inflammatory signaling (31). Collectively, these features allow m^6^A to shape the overall rhythmicity and kinetics of immune responses at the systems level.

## 3 Immune rheostat of innate antiviral immunity

Accumulating evidence suggests that m6A may function as an immune rheostat within innate antiviral immunity by coordinately regulating immune sensing, signal amplification, and inflammatory output (30). Through these regulatory processes, m6A may help maintain a balance between effective antiviral defense and limitation of immunopathology (32). This dynamic modulation allows the innate immune system to maintain a delicate balance between effective defense and tissue homeostasis.

### 3.1 Regulation of PRR activation thresholds: tuning immune sensitivity

Host cells detect viral RNA through pattern recognition receptors (PRRs), among which RIG-I and MDA5 recognize distinct RNA structural features and transmit signals via MAVS to initiate downstream antiviral responses (33). m^6^A modification can modulate the efficiency of PRR-RNA interactions by altering RNA secondary structure stability and conformational dynamics, thereby influencing the “activation threshold” of immune sensing.

Together, these observations suggest that m6A may influence both viral RNA recognition and intracellular signaling capacity, thereby contributing to immune sensitivity (14, 34). In parallel, m^6^A also regulates the stability and translational efficiency of mRNAs encoding PRRs and their downstream signaling components. This dual mode of action enables m^6^A to control both “extrinsic recognition” of viral RNA and “intrinsic tuning” of signaling capacity, thereby establishing a system-level set point for immune activation (34, 35).

Importantly, different RNA viruses exhibit distinct PRR dependencies. For instance, Influenza A virus is primarily sensed by RIG-I, whereas SARS-CoV-2 relies more heavily on MDA5-mediated recognition. This divergence suggests that m6A may differentially modulate RNA structural features to selectively influence the activation thresholds of specific PRRs in a virus-dependent manner (36). However, direct evidence supporting virus-specific rheostatic regulation by m6A in SARS-CoV-2 remains limited.

### 3.2 Control of IFN amplitude and dynamics: coordinating induction and resolution

Following PRR activation, the immune rheostat is further reflected in the quantitative and temporal regulation of type I interferon responses. Type I Interferon represents a central axis of antiviral immunity, requiring precise coordination in both magnitude and timing. Accumulating evidence indicates that m^6^A participates in the full trajectory of IFN responses through multilayered regulatory mechanisms (23, 30).

During the induction phase, m^6^A modulates the stability and translation of key transcripts such as IFNB1, thereby determining the initial output of interferon production. In the amplification phase, m^6^A regulates the expression of critical components in the JAK-STAT pathway, influencing the activation strength of interferon-stimulated genes (ISGs) (30). In the resolution phase, m^6^A promotes the degradation of these transcripts, ensuring timely attenuation of signaling and preventing prolonged immune activation.

In addition, m^6^A may regulate the expression of negative feedback regulators, such as members of the SOCS family, introducing additional control loops into the IFN pathway (37). Through these mechanisms, m^6^A not only determines the peak amplitude of IFN responses but also shapes their temporal kinetics, defining the overall “response curve” of antiviral signaling (38).

Comparatively, Influenza A virus often exhibits stage-specific modulation of IFN responses (39), whereas SARS-CoV-2 tends to delay and suppress interferon activation to facilitate immune evasion. These differences further underscore the context-dependent role of m^6^A in shaping antiviral immune dynamics across distinct viral infections. These comparisons are intended to illustrate potentially conserved regulatory principles and should not be interpreted as direct evidence for SARS-CoV-2.

### 3.3 Inflammatory buffering as a manifestation of the immune rheostat

Building upon the rheostatic regulation of interferon responses described in Section 3.2, m^6^A further contributes to immune homeostasis through inflammatory transcript turnover and cytokine buffering. During the late phase of antiviral immunity, sustained inflammatory activation can drive tissue damage. m^6^A-mediated rheostatic regulation establishes a dynamic inflammatory buffering system, fine-tuning both the magnitude and duration of cytokine expression to prevent immunopathology (40, 41). On the one hand, m^6^A marks promote degradation of pro-inflammatory transcripts such as IL-6 and TNF, limiting their persistence (42, 43). On the other hand, during early immune activation, m^6^A can enhance the translational efficiency of these transcripts to ensure an effective antiviral response (27, 30).

Beyond direct modulation of cytokine mRNAs, m^6^A influences upstream signaling pathways, including components of the NF-κB cascade, thereby modulating both amplification and feedback of inflammatory responses (30, 44). This multilayered control enables the immune system to balance antiviral defense with tissue protection.

Importantly, inflammatory buffering represents a specific manifestation of the broader immune rheostat, demonstrating how m^6^A-mediated rheostatic regulation continuously calibrates immune outputs across infection stages (45). By integrating RNA stability, translational control, and temporal dynamics, the immune rheostat ensures that inflammatory responses are neither insufficient to suppress viral replication nor excessive to cause collateral tissue damage.

Although several components of this regulatory framework have been demonstrated experimentally, many aspects of the proposed immune rheostat remain to be validated directly in SARS-CoV-2 infection models.

## 4 Potential viral exploitation of m^6^A-mediated immune regulation

Although viruses have traditionally been viewed as suppressing host immunity, accumulating evidence suggests that many RNA viruses dynamically modulate immune responses rather than simply inhibiting them. Emerging studies further indicate that m^6^A-mediated RNA regulation may contribute to this process by influencing RNA stability, translation, and immune signaling. While direct mechanistic evidence remains limited for SARS-CoV-2, observations from SARS-CoV-2 together with studies of other RNA viruses support the hypothesis that m^6^A-dependent regulation may represent one mechanism through which viruses fine-tune host immune responses (23).

To provide a SARS-CoV-2-centered overview of current evidence, **Table 1** summarizes representative studies describing m^6^A modification sites and m^6^A-associated regulatory mechanisms reported during SARS-CoV-2 infection. These findings summarize the current SARS-CoV-2-specific evidence available to date and provide the experimental basis for several concepts discussed in this section.

**Table 1:** Representative evidence of m^6^A-mediated regulation during SARS-CoV-2 infection.

Evidence category	SARS-CoV-2 findings	m^6^A regulator(s)	Functional consequence	Experimental model
Viral RNA methylation landscape	Multiple m^6^A-enriched regions identified across genomic and subgenomic RNAs, particularly within ORF1ab, N, and 3′ regions	METTL3/METTL14	Regulates viral RNA metabolism and replication	*In vitro* cell lines
Viral replication	Genetic or pharmacological inhibition of m^6^A machinery reduces SARS-CoV-2 replication	METTL3	Supports efficient viral propagation	*In vitro* cell lines
Viral RNA translation	m^6^A promotes ribosome loading and viral protein synthesis	YTHDF proteins	Enhances translational efficiency of viral RNA	*In vitro* cell lines
Host antiviral response	m^6^A regulates interferon-related transcripts and antiviral signaling pathways	METTL3, FTO, YTHDF family	Modulates IFN production and downstream ISG expression	*In vitro* cell lines
Immune evasion	m^6^A modification may reduce innate immune sensing of viral RNA	Multiple m6A regulators	Potential attenuation of PRR-mediated recognition	*In vitro* cell lines
Reprogramming of host machinery	SARS-CoV-2 infection alters localization and activity of m^6^A regulators	METTL3	Reshapes host–virus epitranscriptomic interactions	*In vitro* cell lines

### 4.1 Modulating rather than silencing immunity

During COVID-19, the host immune response is characterized by a distinctive “delayed activation-sustained inflammation” phenotype, a pattern that is difficult to attribute to inhibition of any single signaling pathway and instead reflects dysregulation at the systems level (46, 47). As a critical regulatory interface linking RNA fate determination with downstream signaling outputs, m^6^A enables viruses to exert multilayered and fine-grained control over host immune responses.

At the early stages of infection, viruses can reshape m^6^A deposition landscapes to reduce the stability or translational efficiency of key antiviral transcripts, including interferons and their upstream signaling regulators (30, 48). Such epitranscriptomic remodeling delays the initiation of innate immune responses, thereby creating a permissive temporal window for viral replication and establishment of infection. At later stages, as viral burden and tissue stress progressively increase, a moderate yet sustained inflammatory state may paradoxically facilitate tissue permeability, immune cell recruitment, and viral dissemination (23, 47). By regulating the persistence and turnover kinetics of inflammation-associated transcripts, m^6^A helps maintain immune activation within a range that is sufficiently restrained to avoid immediate host clearance while remaining permissive for continued viral propagation.

This strategy of “modulation rather than suppression” highlights a sophisticated mode of viral immune exploitation. Rather than functioning through complete immune silencing, viruses appear to utilize m^6^A-dependent regulation to recalibrate host immune activity toward a metastable state that dynamically balances antiviral pressure with replicative fitness (49, 50).

Importantly, although several studies support individual components of this regulatory model, direct evidence demonstrating that SARS-CoV-2 actively establishes an m^6^A-dependent immune rheostat remains limited. Therefore, the mechanisms discussed here should be interpreted as a conceptual framework integrating current experimental observations rather than a fully established model.

### 4.2 Functional roles of m^6^A on viral RNA: from translational enhancement to immune camouflage

Beyond its regulatory effects on host transcripts, m^6^A modification of viral RNA itself has emerged as a critical determinant of viral fitness and host-virus interactions during infection (16, 51). Multiple m^6^A sites have been identified within both genomic and subgenomic RNAs of SARS-CoV-2, with their distribution and abundance exhibiting dynamic changes throughout the course of infection (52).

At the translational level, m^6^A marks can recruit reader proteins such as YTHDF1, thereby enhancing ribosome loading and promoting efficient viral protein synthesis (52, 53). This mechanism provides viral RNAs with a competitive translational advantage, particularly under conditions in which host translational resources are limited or globally repressed during antiviral stress responses. By selectively optimizing translational efficiency, m^6^A enables viruses to sustain productive replication while minimizing the metabolic burden associated with extensive transcriptional reprogramming.

Beyond translation, m^6^A modification may also influence immune recognition of viral RNA. By altering RNA secondary structure or masking immunostimulatory motifs, m^6^A can reduce the accessibility of viral RNA to pattern recognition receptors (PRRs), thereby attenuating innate immune sensing (14). This phenomenon can be conceptualized as “RNA camouflage,” in which viral transcripts acquire a more “self-like” molecular identity that facilitates evasion of immune surveillance. For example, an m^6^A site within the N gene at position 28,144 (within a DRACH motif) has been experimentally shown to reduce RIG-I binding while enhancing YTHDF1-mediated translation of the N transcript (23). Similarly, multiple m^6^A modification sites have been identified in the ORF3a coding region of SARS-CoV-2 RNA through direct nanopore sequencing, suggesting that epitranscriptomic marks in this locus may influence transcript stability and immune recognition by host antiviral pathways (54). Unlike fixed genomic mutations, however, epitranscriptomic camouflage is inherently reversible and dynamically regulated, enabling viruses to fine-tune their immunogenicity according to distinct stages of infection and host cellular states.

**Figure 2:**
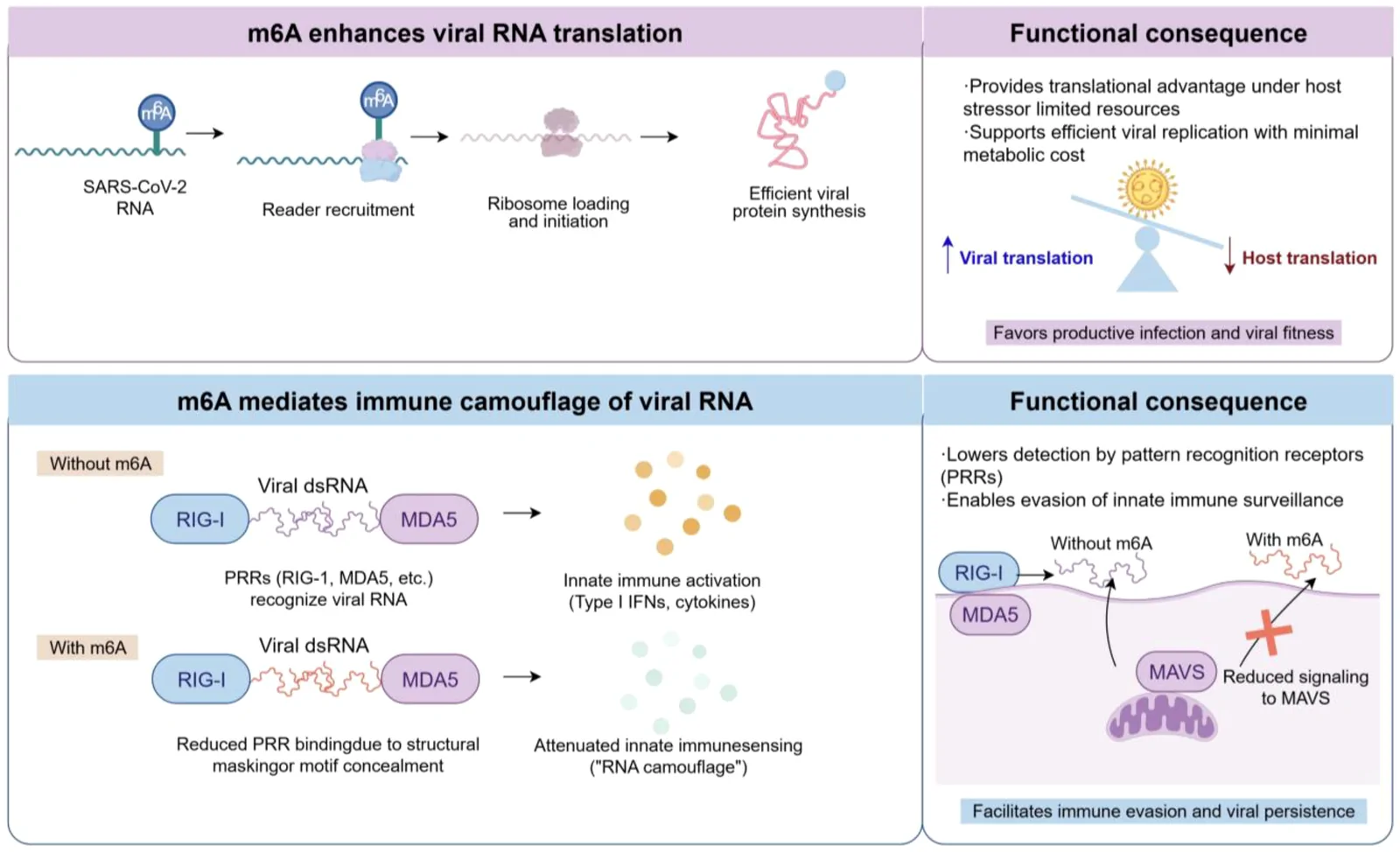
m^6^a on viral RNA: translational enhancement and immune camouflage. This image illustrates the role of m6A in regulating SARS-CoV-2 RNA fate. m6A enhances viral RNA translation by promoting ribosome loading and increases viral protein production, while simultaneously reducing innate immune recognition by masking viral RNA from PRR sensing. Together, these effects support efficient viral replication and immune evasion.

Importantly, the effects of m^6^A on viral RNA are highly context-dependent rather than uniformly pro-viral. Depending on cell type, metabolic state, and infection stage, m^6^A may differentially balance translational enhancement with modulation of immune visibility (55, 56). Notably, some mechanistic interpretations regarding the dynamic interplay between m^6^A-mediated translational regulation and immune recognition are extrapolated from studies of other RNA viruses and remain to be experimentally validated in the context of SARS-CoV-2 infection. This dual functionality further reinforces the concept that m^6^A operates not as a static viral accessory mechanism, but as a rheostatic regulatory layer capable of continuously tuning the equilibrium between viral replication and host immune detection (**Figure 2**).

### 4.3 Reprogramming host m^6^A machinery: from local modulation to system-level remodeling

Beyond directly modifying viral and host RNA substrates, SARS-CoV-2 infection has been reported to alter reprogram host m^6^A machinery to exert indirect yet system-wide control over immune regulation. During infection with SARS-CoV-2, redistribution of METTL3 from the nucleus to the cytoplasm has been observed, resulting in altered substrate preferences between viral RNA and host transcripts (57). This spatial reorganization enables selective reallocation of m^6^A marks across distinct RNA populations, thereby reshaping the epitranscriptomic landscape at a transcriptome-wide scale. Rather than acting on isolated targets, such remodeling allows viruses to coordinately adjust multiple layers of host gene regulation simultaneously.

Concomitantly, both the localization and functional properties of m^6^A reader proteins undergo dynamic remodeling during infection. Members of the YTHDF protein family can accumulate within stress granules, P-bodies, or RNA decay complexes, thereby altering transcript selectivity and facilitating functional switching between translational enhancement and mRNA degradation (58, 59). By regulating the availability and subcellular distribution of reader proteins, viruses indirectly determine how m^6^A marks are “interpreted,” ultimately reshaping the expression programs of immune-related genes. This layer of regulation introduces substantial plasticity into antiviral signaling networks and allows immune outputs to be continuously adjusted according to infection stage and cellular context.

Importantly, this regulatory strategy extends beyond individual signaling pathways to encompass the broader RNA regulatory network. Through reconfiguration of m^6^A machinery, viruses effectively reconstruct a global regulatory architecture that coordinately impacts multiple immune nodes, including pathogen sensing, signal amplification, interferon responsiveness, and inflammatory output (30). Compared with direct inhibition of discrete signaling molecules, such systems-level remodeling is both metabolically efficient and mechanistically less conspicuous, enabling viruses to manipulate host immunity while minimizing overt disruption of cellular homeostasis.

Comparable patterns have also been reported in other RNA viruses. For example, Influenza A virus infection is likewise associated with alterations in m^6^A deposition patterns and redistribution of m^6^A regulatory factors (22), suggesting that m^6^A-mediated immune modulation may represent a conserved adaptive strategy shared among diverse RNA viruses. Although the precise molecular mechanisms differ between viral species, a common principle emerges in which viruses indirectly reshape immune responses through modulation of RNA fate decisions, thereby maintaining a dynamic equilibrium between replicative fitness and immune evasion.

Collectively, viral exploitation of the m^6^A system appears to operate through three interconnected layers: (i) fine-tuning the magnitude and kinetics of host immune transcripts; (ii) modifying viral RNA to optimize translation while reducing immune visibility; and (iii) reprogramming host m^6^A machinery to remodel immune regulatory networks at a systems level. Although direct evidence remains incomplete for SARS-CoV-2, these observations provide a conceptual framework for understanding how m^6^A-dependent regulation could influence the balance between viral replication and host immunity. It should be emphasized that many mechanistic links discussed in this section are inferred from studies of Influenza A virus, flaviviruses, or general m^6^A biology. Although SARS-CoV-2-specific studies have identified viral m^6^A sites and alterations in m^6^A regulators, direct evidence demonstrating coordinated rheostatic regulation across infection stages remains limited. Future studies combining transcriptome-wide m^6^A profiling with functional perturbation in physiologically relevant SARS-CoV-2 models will be required to test this proposed framework.

## 5 Integration with immunometabolism

During viral infection, immune responses and cellular metabolism are not independent systems but are tightly interconnected through multilayered signaling networks (60, 61). Increasing evidence indicates that metabolic states not only dictate immune cell function but are themselves dynamically reshaped by antiviral signaling pathways (62, 63). Within this bidirectional regulatory framework, m^6^A modification has emerged as a critical molecular hub linking metabolic reprogramming to immune effector functions through post-transcriptional regulation of gene expression (18, 19). In the context of SARS-CoV-2 infection, emerging evidence suggests that this coupling may become particularly important, whereby RNA-level regulation potentially links metabolic flux alterations to dynamic immune outputs.

We propose that immunometabolic coupling represents a key dimension of the m^6^A-mediated immune rheostat. Beyond directly regulating immune transcripts, m^6^A may fine-tune antiviral immunity by reshaping metabolic programs that influence immune cell activation and inflammatory outputs. Thus, metabolic reprogramming can be viewed not as an independent function of m^6^A, but as an integral mechanism through which the m^6^A rheostat modulates the magnitude, duration, and quality of antiviral immune responses. Importantly, while increasing evidence supports individual components of the m^6^A–metabolism–immunity axis, direct experimental validation of this integrated rheostatic model in SARS-CoV-2 infection remains limited.

### 5.1 m^6^A regulation of metabolic enzyme expression: driving metabolic reprogramming

Accumulating evidence indicates that m^6^A may reshape intracellular metabolic flux by modulating the stability and translational efficiency of mRNAs encoding metabolic enzymes, thereby reprogramming the metabolic phenotype of immune cells (18, 19).

In glycolytic pathways, the expression and translation of rate-limiting enzymes such as HK2, PFK, and LDHA are subject to m^6^A-dependent regulation (64, 65). By enhancing transcript stability and translational efficiency, m^6^A has been reported to promote glycolytic flux, facilitating rapid ATP production and generation of biosynthetic intermediates required for cytokine synthesis and antiviral responses. Such a glycolytic shift represents a hallmark metabolic adaptation during the early phase of viral infection and immune activation (35, 66).

Within lipid metabolic networks, m^6^A has been implicated in regulating enzymes involved in fatty acid synthesis and β-oxidation, including FASN, ACC, and CPT1, thereby influencing lipid droplet biogenesis and membrane remodeling (62, 67). Lipids not only provide structural components essential for viral replication, such as viral envelope formation, but also modulate immune signaling by shaping membrane microdomains involved in receptor organization and downstream signal transduction (68, 69). Consequently, m^6^A-mediated lipid metabolic reprogramming may simultaneously support viral replication and remodels the immune microenvironment.

m^6^A also interfaces with one-carbon metabolism through regulation of enzymes responsible for the generation of methyl donors such as S-adenosylmethionine (SAM) (70, 71). This interaction is particularly significant because m^6^A deposition itself depends on cellular methyl donor availability, thereby establishing a bidirectional feedback loop in which metabolic state influences m^6^A dynamics, while m^6^A reciprocally regulates metabolic enzyme expression. Such “metabolism–epitranscriptome coupling” constitutes a self-reinforcing regulatory circuit that integrates nutrient sensing with immune adaptation.

During SARS-CoV-2 infection, extensive metabolic remodeling has been reported, including enhanced glycolysis, altered lipid metabolism, and perturbation of one-carbon metabolic pathways. These metabolic changes are closely associated with viral replication and dysregulated immune responses (72, 73). Emerging evidence further suggests that m^6^A regulators, particularly METTL3, may participate in coordinating these metabolic adaptations, although the precise transcript-specific mechanisms remain incompletely defined. Most mechanistic evidence linking m^6^A-dependent metabolic regulation to antiviral immunity has been obtained from cancer biology or other viral infection models, and its applicability to SARS-CoV-2 remains to be fully established.

Collectively, through coordinated regulation of glycolysis, lipid metabolism, and one-carbon metabolism, m^6^A drives the transition from homeostatic metabolism toward an “immune-activated metabolic state,” thereby providing the energetic and biosynthetic resources required to sustain antiviral immunity, consistent with its role as a rheostatic regulator of immune outputs.

### 5.2 Metabolic feedback on immune responses: from energy supply to signal modulation

Metabolic reprogramming is not merely a downstream consequence of immune activation but also a critical determinant of immune signaling outputs (60). By reshaping cellular metabolic states, m^6^A may indirectly modulate the magnitude and duration of immune responses, thereby establishing a bidirectional regulatory circuit between metabolism and immunity.

At the level of bioenergetics, enhanced glycolysis provides rapid ATP production to sustain immune signaling, protein synthesis, and cytokine secretion. For example, the production and release of Type I Interferons are highly dependent on metabolic availability, and m^6^A-driven upregulation of glycolytic enzymes helps support these energetic demands (35, 74). Conversely, metabolic restriction or impaired glycolytic flux can attenuate interferon signaling and compromise antiviral defense mechanisms.

In COVID-19, excessive glycolytic activation and accumulation of metabolic intermediates have been associated with aberrant inflammatory responses and impaired antiviral immunity. Metabolic products such as lactate can suppress antiviral signaling pathways, whereas lipid-derived mediators may contribute to sustained inflammation. These observations raise the possibility that m^6^A-dependent regulation of metabolic pathways may influence immune outcomes during SARS-CoV-2 infection by controlling the availability of key metabolites and energy resources.

Beyond energy supply, metabolic intermediates themselves function as immunoregulatory signaling molecules (75). Lactate accumulation, for instance, has been shown to suppress antiviral signaling pathways, whereas lipid-derived metabolites can potentiate inflammatory responses (62). By regulating key metabolic nodes, m^6^A indirectly influences the abundance of these intermediates and thereby modulates immune signaling at a higher regulatory level. In parallel, one-carbon metabolism controls the availability of methyl donors required for both epigenetic and epitranscriptomic modifications, further amplifying the regulatory influence of m^6^A on immune responses (70).

Collectively, metabolic states not only provide the energetic foundation for immune activation but also actively shape signaling outputs, with m^6^A functioning as a central intermediary that integrates metabolic inputs into coordinated immune responses, reinforcing its role as a rheostatic regulator that fine-tunes antiviral immunity.

### 5.3 A three-layer regulatory network: from RNA modification to immune output

**Figure 3:**
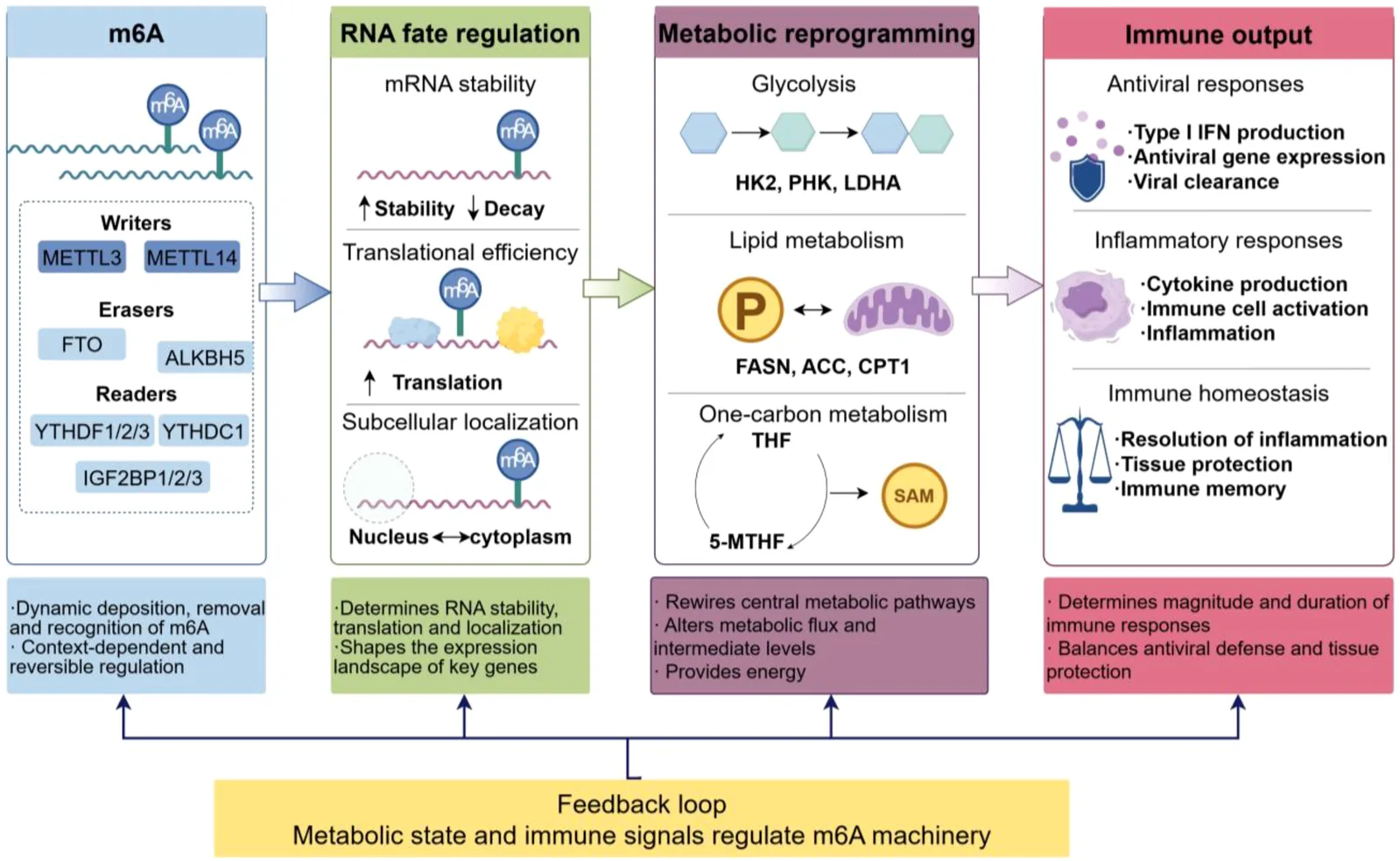
A three-layer regulatory network: from RNA modification to immune output. This image summarizes a three-layer regulatory network linking m^6^A RNA modification to immune outcomes during viral infection. The top layer describes m^6^A regulators controlling RNA fate, the middle layer illustrates how m^6^A reshapes RNA stability, translation, and cellular metabolic pathways, and the bottom layer shows how these changes ultimately tune antiviral and inflammatory immune responses through a feedback loop between metabolism, immunity, and the m^6^A machinery.

Integrating these mechanisms, the role of m^6^A in immunometabolic regulation can be conceptualized as a three-layer regulatory network (As shown in **Figure 3**):

In this framework, m^6^A is proposed to act as an upstream regulatory node that shapes the expression landscape of metabolic enzymes through the control of RNA stability and translation (76). Metabolic reprogramming constitutes the intermediate layer, converting molecular alterations into redistributed metabolic flux. Ultimately, metabolic states may influence metabolic states influence immune outputs by modulating energy availability and signaling metabolites, thereby determining the magnitude, duration, and functional characteristics of immune responses (77, 78). This model emphasizes that antiviral immunity is not governed by isolated signaling pathways but instead emerges from coordinated, multilayered network interactions. Similar metabolic reprogramming phenomena have been observed in other RNA virus infections. For example, Influenza A virus infection also induces enhanced glycolysis and lipid remodeling, suggesting that metabolism–immunity coupling represents a conserved feature of RNA virus-host interactions, with m^6^A likely serving as a critical regulatory node (79, 80). Overall, m^6^A not only directly regulates immune-related transcripts but also indirectly shapes immune responses through higher-order reprogramming of cellular metabolism. Its central position within the “metabolism–immunity axis” enables the integration of diverse signals into coordinated immune outputs, reinforcing its role as a key regulatory hub in antiviral host defense.

Nevertheless, direct evidence linking specific m^6^A-regulated metabolic transcripts to immunometabolic remodeling during SARS-CoV-2 infection remains limited. Therefore, some aspects of the proposed m^6^A–metabolism–immunity regulatory axis are currently inferred from studies in other viral systems or related biological contexts and require further experimental validation in SARS-CoV-2 infection.

## 6 Therapeutic targeting: resetting the immune rheostat

Viewing m^6^A as an “immune rheostat” provides a critical conceptual shift for antiviral intervention: the goal should not be simply to enhance or suppress immunity, but to precisely recalibrate its magnitude and temporal dynamics (81). This perspective is particularly relevant in COVID-19 caused by SARS-CoV-2, where disease progression is characterized by a dynamic imbalance of “early insufficiency followed by late hyperactivation (82).”

### 6.1 Targeting the m^6^A machinery: from individual enzymes to regulatory networks

Current therapeutic strategies targeting the m^6^A system primarily focus on key enzymatic components. The methyltransferase METTL3, as the core “writer,” represents a central node whose inhibition can globally alter m6A deposition and consequently reshape the expression landscape of immune-related transcripts (83, 84). In principle, during phases of excessive inflammation, partial inhibition of METTL3 activity may reduce the translation or persistence of pro-inflammatory mediators, thereby mitigating immunopathology (85).

Conversely, the demethylase FTO removes m^6^A marks and modulates transcript stability (86). Inhibition of FTO may, in certain contexts, prolong the expression of antiviral genes or restore immune responses suppressed by viral infection (87). Such an approach could be particularly advantageous in the early stages of infection, where strengthening the baseline antiviral response is critical.

**Figure 4:**
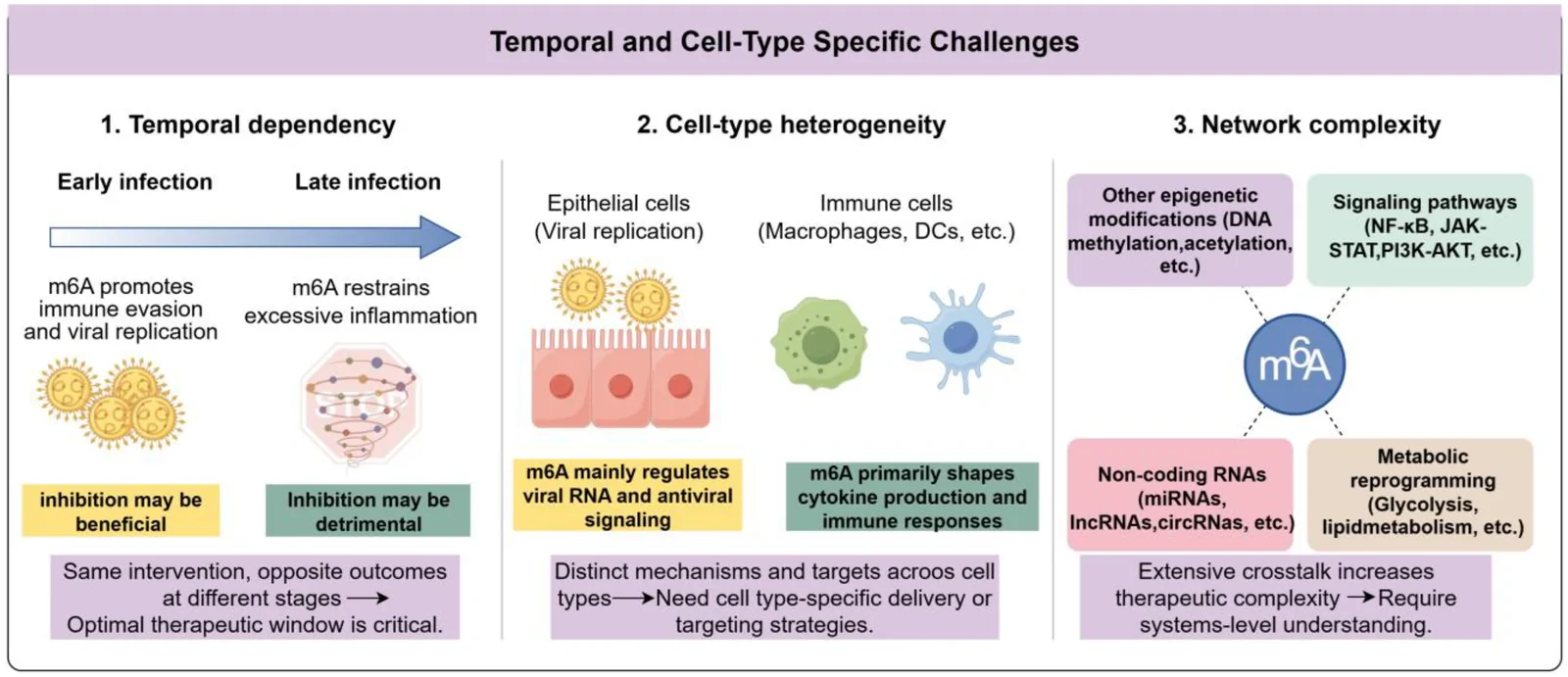
Temporal and cell-type specific challenges. This image summarizes the major challenges in m^6^A-mediated regulation during viral infection, highlighting its strong temporal and cell-type specificity. The figure illustrates how the effects of m^6^A vary between early and late stages of infection, differ across epithelial and immune cells, and are further complicated by extensive crosstalk with epigenetic modifications, signaling pathways, non-coding RNAs, and metabolic reprogramming.

Several small-molecule modulators targeting the m^6^A machinery have recently been developed, including the METTL3 inhibitor STM2457 and FTO inhibitors such as FB23-2, CS1, and CS2 (83, 88). Although these compounds exhibit promising activity in preclinical models of cancer and inflammatory disorders, evidence supporting their antiviral efficacy remains limited. While emerging studies suggest that manipulation of m^6^A regulators can influence viral replication and host antiviral responses, systematic evaluation in SARS-CoV-2 infection models is still lacking. Moreover, the effects of these compounds on antiviral immunity, viral fitness, and host toxicity remain incompletely understood. Therefore, despite their therapeutic potential, all current m^6^A-targeting strategies remain at the preclinical proof-of-concept stage and are still far from translational application (**Figure 4**).

However, it is important to recognize that the m^6^A machinery operates as a broad and interconnected regulatory system. Its targets extend beyond immune-related transcripts to include genes involved in metabolism, cell cycle control, and stress responses (89). Consequently, global perturbation of a single component may produce off-target or systemic effects. Future strategies with greater translational potential may involve selective modulation of specific reader proteins or the development of RNA-targeting technologies capable of editing m^6^A marks at defined transcript sites, thereby achieving higher precision in immune regulation (90).

### 6.2 Therapeutic logic: from “enhancing immunity” to “recalibrating immune intensity”

Conventional antiviral immunotherapies are often centered on enhancing immune responses, such as boosting interferon production or activating inflammatory pathways (91). However, in diseases like COVID-19, the fundamental issue lies in the loss of regulatory precision rather than absolute immune deficiency (2). Accordingly, m^6^A-targeted strategies should aim to recalibrate the intensity and dynamics of immune responses.

Specifically, during the early phase of infection, enhancing m^6^A-mediated translational control may accelerate the expression of antiviral genes, thereby improving viral clearance (92). In contrast, during later stages, promoting the degradation or translational repression of inflammation-related transcripts may limit excessive immune activation and prevent tissue damage (93). In other words, m6A-based interventions should dynamically shift between “enhancement” and “attenuation” depending on disease stage, enabling bidirectional control of immune responses.

The central premise of this strategy is to treat the immune system as a continuously adjustable dynamic system rather than a binary switch. By targeting m^6^A as a key regulatory node, it becomes possible to reshape the overall immune response curve-modulating its peak amplitude, onset timing, and duration at the molecular level (94).

### 6.3 Temporal and cell-type specific challenges

Despite its therapeutic promise, m^6^A-targeted intervention faces significant challenges, particularly with respect to temporal dependency and cellular heterogeneity (95).

First, the functional role of m^6^A varies across different stages of infection. For example, in early infection, m^6^A may contribute to suppression of antiviral signaling, facilitating viral replication, whereas in later stages, it may act to constrain inflammation and prevent immunopathology (23, 30). As a result, the same therapeutic intervention could produce opposing effects depending on timing, underscoring the importance of identifying optimal therapeutic windows.

Second, the dependence on m^6^A regulation differs among cell types. Respiratory epithelial cells primarily support viral replication, while immune cells such as macrophages and dendritic cells orchestrate immune responses (96). The mechanisms and targets of m^6^A regulation in these cell populations are distinct, meaning that systemic interventions may fail to achieve desired outcomes (93). The development of cell type–specific delivery systems or targeting strategies will therefore be essential to improve therapeutic precision (97).

Finally, the m^6^A regulatory network exhibits extensive crosstalk with other epigenetic and signaling pathways, further increasing the complexity of therapeutic manipulation (98). A comprehensive, systems-level understanding of m^6^A dynamics across different cell types and temporal stages will be crucial for advancing its clinical translation.

## 7 Conclusions and perspectives

Collectively, the evidence discussed in this review supports the development of a hypothesis-driven conceptual framework for understanding the role of m^6^A in antiviral immunity. Rather than functioning as a simple binary switch, m^6^A may act as a tunable regulatory layer that continuously shapes immune responses through its control over RNA fate. By coordinating transcript stability, translational efficiency, and expression kinetics, m^6^A has the potential to modulate immune signaling across multiple dimensions. Accordingly, we propose that m^6^A can be conceptualized as an epitranscriptomic immune rheostat, capable of fine-tuning the balance between effective antiviral defense and the prevention of immunopathology.

Current evidence suggests that, during SARS-CoV-2 infection, m^6^A-mediated regulation may influence multiple layers of antiviral immunity, including pathogen sensing, interferon signaling, inflammatory responses, and metabolic adaptation. At the level of pathogen recognition, m^6^A has been implicated in modulating the activation thresholds of pattern recognition receptors. During downstream signaling, it may contribute to the magnitude and kinetics of type I interferon responses. In later stages of infection, m^6^A has been reported to influence cytokine transcript stability and translation, potentially limiting excessive inflammatory responses. Beyond canonical immune pathways, emerging evidence further suggests that m^6^A may participate in coordinating metabolic reprogramming with immune function. Although these findings support an integrated regulatory model, direct experimental validation of the proposed immune rheostat in SARS-CoV-2 infection remains incomplete.

Despite these advances, several key challenges remain. First, a comprehensive understanding of m^6^A dynamics at single-cell resolution is still lacking. The integration of single-cell sequencing with spatial transcriptomics will be essential to map the heterogeneity of m^6^A regulation across different cell types and infection stages. Second, most current studies focus on global changes in m^6^A levels, whereas the functional significance of site-specific modifications remains insufficiently resolved. The development of precise RNA editing tools, including CRISPR-based and programmable RNA-targeting systems, will enable targeted manipulation of individual m^6^A sites and provide deeper mechanistic insights.

In addition, the interplay between m^6^A and other post-translational modifications represents an emerging frontier. For example, m^6^A-mediated regulation of RNA stability may intersect with ubiquitin-dependent protein degradation pathways, forming coordinated or antagonistic regulatory circuits in antiviral signaling. Such multilayered “RNA-protein” regulatory crosstalk offers a promising avenue for understanding the complexity of virus-host interactions. Whether these regulatory interactions contribute to the proposed immune rheostat remains an important question for future investigation.

Looking forward, continued investigation of the m^6^A regulatory network may facilitate the development of next-generation host-directed antiviral strategies. Rather than directly validating the immune rheostat model, future studies should determine whether precise manipulation of m^6^A can quantitatively and temporally recalibrate antiviral immunity in physiologically relevant infection models. Such studies will be critical for evaluating the translational relevance of the proposed framework. Although m^6^A-targeting interventions hold considerable promise, current therapeutic strategies remain at the preclinical proof-of-concept stage, and systemic modulation of m^6^A may carry risks of broad off-target effects because of its fundamental roles in cellular physiology. Therefore, future translational efforts should prioritize specificity, safety, and context-dependent targeting.

Overall, the concept of the epitranscriptomic immune rheostat should be viewed as a testable conceptual framework that integrates current evidence and provides a foundation for future mechanistic and translational studies, rather than as a fully established model of SARS-CoV-2 pathogenesis.
